# Spatial Distribution and Multilevel Analysis of Pregnancy Loss in India: Examining Individual and Contextual Factors

**DOI:** 10.34172/jrhs.2025.180

**Published:** 2025-04-01

**Authors:** Mahadev Bhise, Sharyu Mhamane, Ranjan Kumar Prusty, Shahina Begum

**Affiliations:** ^1^Department of Biostatistics, Indian Council of Medical Research, National Institute for Research in Reproductive and Child Health, Mumbai, India; ^2^Centre for Cancer Epidemiology (CCE), Tata Memorial Centre (TMC), Navi Mumbai, Maharashtra, India; ^3^Faculty of Medical Research, Academy of Scientific Innovation and Research (AcSIR), Ghaziabad, Uttar Pradesh, India

**Keywords:** Pregnancy, Stillbirths, Miscarriages, Abortions, Spatial analysis, India

## Abstract

**Background:** Around 810 women die daily due to adverse pregnancy outcomes (APOs), predominantly in low- and middle-income countries. In India, despite advancements in maternal health initiatives, pregnancy loss (PL) rates remain high. This study analyzed the determinants, prevalence, and spatial distribution of PL in India.

**Study Design:** This study employed a cross-sectional design.

**Methods:** Using data from the National Family Health Survey (NFHS-5, 2019-2021), the study analyzed 255,385 pregnancies to assess the prevalence of PL. The analysis includes socio-demographic variables and spatial factors affecting PL rates.

**Results:** The national PL prevalence is 11.1%, comprising 7.3% miscarriages, 2.9% abortions, and 0.9% stillbirths. Higher PL rates correlated with older maternal age, urban residence, higher wealth index, and tobacco use. The spatial analysis identified 84 districts as hot spots for PL, primarily located in Northern and Eastern India, while 89 cold spots were identified in Central and North-Eastern regions. Multilevel logistic regression revealed that women aged 35-49 years (aOR=3.8, 95% CI: 1.26-1.63) and women who used tobacco (aOR: 1.28, 95% CI: 1.09-1.49) were at a significantly higher risk of PL compared to younger women (<20 years) and non-tobacco users, respectively.

**Conclusion:** The study highlights the need for further research to elucidate the underlying causes of PLs and recommends strengthening the health system in hot spot districts. This can be achieved through targeted interventions that address regional disparities and socio-economic determinants, ultimately improving maternal health outcomes.

## Background

 Pregnancy is a crucial phase in any woman’s life, requiring utmost care and attention. Failure to ensure proper care during this period leads to a greater risk of adverse pregnancy outcomes (APOs). APOs encompass a wide range of health problems that affect the mother, child, or both during pregnancy, labor, delivery, or the postpartum phase. These include stillbirth, low birth weight, hypertensive disorders during pregnancy, obstructed labor, and antepartum/postpartum hemorrhage.^1^

 Miscarriage and stillbirth are the most common natural pregnancy losses (PLs), affecting the mother’s physical and psychosocial well-being.^2^ India is one of the six countries that share half the global burden of stillbirths, and the causes remain unexplained for one-third of cases.^3,4^ The American College of Obstetricians and Gynecologists estimates that abortion accounts for 26% of PLs, which is the most common form, followed by miscarriages, contributing up to 10% of clinically perceived pregnancies.^5^

 According to the World Health Organization (WHO), nearly 810 women die daily due to APOs every day, with 94% of these deaths occurring in low- and middle-income countries, 66% in sub-Saharan Africa and 20% in South Asia. In developing countries like India, approximately 44,000 deaths from adverse pregnancy-related outcomes occur each year.^6^ While APOs occur frequently in developing countries, they pose a public health concern in both developed and developing countries.^7^

 Existing literature identifies a plethora of factors that influence the occurrence of PL, including maternal age,^8-10^ wealth index,^8,10,11^ excessive work,^8,11^ place of residence, maternal education, religion, Body Mass Index, anemia level,^8^ spousal violence,^12^ alcohol or liquor consumption during pregnancy,^8,13^ lack of antenatal care visits.^14^ Gestational diabetes mellitus is also an increasingly prevalent clinical complication that adversely impacts pregnancy.^15^

 Although PL is widely studied in developed and developing countries,^16,17^ research on its trends and patterns remains scarce from an Indian perspective. Literature suggests that PL prevalence is higher in India due to higher rates of APOs.^7,8,10^ APOs pose a serious threat to the lives of both the mother and child, making it a critical public health issue. In recent years, India has made remarkable strides to improve Reproductive, Maternal, Neonatal, Child, and Adolescent (RMNCH + A) health. Some of these maternal and child health (MCH) interventions include Janani Suraksha Yojana (2005), Pradhan Mantri Surakshit Matritva Abhiyan (2016), Pradhan Mantri Matru Vandana Yojna (2017), and LaQshya (2017). These programs aim to provide high-quality free antenatal care during delivery, identify high-risk pregnancies, and offer cash incentives.^18,19^

 Despite the availability of such interventions, challenges remain in the uptake of available services, particularly among underprivileged and socio-culturally diverse populations who have poor utilization rates of the maternal health services.^11^ These gaps are evident due to the urban-rural divide and worsen further among tribal populations.^11^ Therefore, it is crucial to understand the determinants and spatial distribution of PLs in India. Spatial analysis is a technique that helps explore the variability and spatial dependence in the relationship between individual-level factors, contextual factors, and PL in India. This study aimed to identify the determinants, prevalence, and spatial distribution of PL in India using data from the National Family Health Survey (NFHS-5) conducted in 2019-2021, which is the latest round of the Indian Demographic Health Survey. This approach enables us to examine how neighboring geographic units may influence observed associations and assess the variability of these associations across different geographic regions. Spatial models will help identify areas where policies and interventions can be prioritized to improve health outcomes, particularly with respect to PL.

## Methods

###  Study design and participants

 The fifth round of the NFHS conducted in 2019-2021 is the latest round of the Indian Demographic Health Survey. NFHS is a cross-sectional survey that collects data from a large sample of ever-married women aged 15-49 across India, covering various health and demographic aspects. The survey is a nationally representative sample, covering all states and union territories of India. Data were collected from 724 115 ever-married women and 636 699 households.^20^ The fourth round of the NFHS conducted during 2015-2016 used similar design and methodology. Detailed information about the NFHS survey, including its sample design and data collection methods, can be found in the NFHS report.^20^

 The NFHS survey also collects calendar data on key life events of the respondents. This calendar records information on various activities and important events in the respondents’ lives such as births, pregnancies, terminations, and contraceptive use. The survey provides a comprehensive history of women’s reproduction and contraceptive use for the 5 to 7 years prior to the survey. The calendar data consists of a matrix of rows and columns, containing the history of events in women’s lives.^20^ In this study, we used the calendar data from the women’s file and birth history file to examine the relationship between individual and community risk factors and PL.

###  Procedures

 Calendar data from the NFHS survey were used to calculate the rates of PL such as stillbirth, abortion, and miscarriage. The analysis was restricted to pregnancies among women aged 15-49 during the five years prior to the survey. The total sample for the study includes 255 385 pregnancies, of which 228 788 are live births, and 26 597 are PL (e.g., stillbirths, miscarriages, and abortions) among ever-married women during the five years preceding the survey.

###  Outcome variable

 The primary dependent variable in this study is PL, defined as pregnancies ending in non-live births. PL is defined as the loss of a fetus within the five years preceding the survey due to adverse events such as stillbirth, miscarriage, or abortion. Stillbirth refers to a late PL that occurs after seven months of gestation. Miscarriage refers to the sudden loss of a pregnancy before 20 weeks of gestation. Abortion refers to the voluntary termination of a pregnancy, whether spontaneous or induced.^21^ For this analysis, we recoded the PL variable into a binary format, with “1” assigned for PL due to any adverse event (e.g., stillbirth, miscarriage, or abortion), and “0” assigned for live births.

###  Independent variables

 The individual and household-level factors include woman’s age at the end of pregnancy, which is re-coded in three categories: < 20, 20-34, and 35-49 years, parity (one, two, and three or more), place of residence (rural/urban), occupation (currently working, categorized as yes/no), wealth status with three categories (poor, middle, rich), and behavioral risk factors such as smoking and alcohol use (yes/no).

 The wealth index is calculated based on information regarding the household’s amenities and assets, as reported by the household respondents. This includes factors such as the main source of drinking water, type of water facility, fuel used for cooking, source of lighting, type of household structure, land ownership status, and possession of various household assets. Using this data, a wealth index was constructed. Each response was assigned a weight, determined through principal component analysis. The score is further converted into percentiles and divided into five categories, with each group representing a 20% segment of the distribution. The lowest two quintiles (poorest and poorer) are grouped and labeled as “poor,” the middle quintile is designated as “middle,” and the top two quintiles (richer and richest) are categorized as “rich.”

 Contextual factors include exposure to mass media, which is measured by the frequency of watching television, reading newspapers or magazines, and listening to the radio. This exposure is categorized into three levels: no exposure, partial exposure, and full exposure. The community factors considered were community education, the Ethnic Fractionalization Index, and the Religious Fractionalization Index.

 Community-level education is indicated by the proportion of women in the community with secondary and higher education, categorized as high or low. The Ethnic and Religious Fractionalization Indices measure the ethnic and religious diversity within the community. These indices are calculated as:

*EFL = 1- Σi (proportion of ethno-linguistic group/religious group ‘i’ in the population)*

 A homogenous category shows no diversity, while a non-homogenous category indicates complete diversity. Detailed calculations are provided elsewhere.^22^

###  Prevalence of pregnancy loss 

 The prevalence of PL across different districts and various background characteristics is measured and presented as percentages. To determine the association between predictor variables and PL, a chi-square test was conducted at a 5% level of significance.

###  Spatial pattern of pregnancy loss 

 The present study aimed to examine the spatial patterns and clustering of PL across districts in India. Geospatial techniques such as Moran’s I and the Local Indicator of Spatial Association (LISA) were utilized to investigate spatial autocorrelation and heterogeneity in the prevalence of PL. Moran’s I is a useful measure to quantify spatial autocorrelation within a dataset across a geographical area. Its values range from -1 to + 1, with positive values close to + 1 indicating spatial clustering, a value of zero indicating spatial randomness, and values close to -1 suggesting dispersion.^23,24^

 Additionally, the Univariate LISA was employed to assess spatial autocorrelation between specific geographical units and their neighbors, thereby identifying the spatial distribution of PL in India in terms of hot spots and cold spots. Spatial autocorrelation occurs when values for a random variable cluster together, whereas negative spatial autocorrelation is observed when a region is surrounded by neighbors with markedly different values.^23,24^ The queen contiguity method, based on shared vertices and boundaries, was employed to identify neighboring regions. A weight matrix was created using this method, assigning values of 1 to neighbors and 0 to non-neighbors, respectively.^24^ Further details on the spatial analysis methodology can be found elsewhere.

 In this study, two types of spatial maps were used to illustrate the distribution of PL: the cluster map and the significance map.

• Cluster map: This map shows the clustering or geographical variation in the prevalence of PL, identifying hot spots and cold spots.
✓ *High-high category (hot spots):* Districts with a high prevalence of PL, surrounded by neighboring districts with similarly high prevalence.
✓ *Low-low category (cold spots): *Districts with a low prevalence of PL, surrounded by neighboring districts with similarly low prevalence.
✓ *High-low category:* Districts with a high prevalence of PL, surrounded by neighboring districts with low prevalence.
✓ *Low-high category*: Districts with a low prevalence of PL, surrounded by neighboring districts with high prevalence.
• Significance map: This map shows Moran’s I statistics in terms of LISA significance. Districts with significant spatial autocorrelation are shaded in green, with different colors in the cluster map highlighting these significant districts.

Both the cluster map and the significance map illustrate the spatial prevalence of PL at a 5% level of significance.

###  Multilevel analysis

 We employed two-level mixed-effect multilevel logistic regression analyses to identify risk factors for PL in India. The fixed part of the model measured risk factors at two levels: individual and district. The random part of the model assessed the random effects or clustering at the district level. Two separate multilevel logistic regression models were constructed:

- Empty model: This model contained no exposure variables and focused solely on decomposing the total variance into district levels, helping to measure the extent of cluster variation in the prevalence of PL.
- Adjusted model: This model included individual and contextual variables.

The results for the fixed effects model are presented as adjusted odds ratios (aORs) with 95% confidence intervals after controlling all factors mentioned in the independent variable section. The advantage of multilevel logistic regressions is their ability to partition the variation in the dependent variable, measured at the individual level, and attribute it to differences among individuals and districts in terms of variance partition coefficient (VPC). Further details on the spatial analysis methodology can be found elsewhere.^22,25^ All statistical analyses for this study were performed using STATA, while spatial analyses were conducted using ArcGIS and GeoDa software.

## Results

###  Percentage distribution of study sample

 The sample distribution according to socio-demographic characteristics is presented in Table 1. About 83.6% of the women belonged to the age group of 20 to 30 years. Around 39% of the women had two children (parity). A significant proportion (72.6%) came from rural areas, and 78% were not currently working. More than two-thirds of the women belonged to the low economic wealth status group. Approximately 65% had partial exposure to mass media, and around 3% of the women consumed tobacco. About 28% of the women belonged to the central region of India, and 74% of the women had high community-level education. Ethnically, 81.7% belonged to heterogeneous ethnic groups, while 65% belonged to completely homogeneous religious groups.

**Table 1:** Percentage distribution of study sample by socio-demographic profile, India 2019-2021

**Background variables**	**Percent**	**Number**
Age at the end of pregnancy (y)		
< 20	12.6	29,184
20-34	83.6	214,354
35-49	3.8	11,847
Parity		
One	27.6	67,921
Two	39.1	95,802
Three +	33.3	87,078
Place of residence		
Rural	72.6	201,906
Urban	27.4	53,479
Working status		
No	78.4	29,453
Yes	21.6	9605
Wealth status		
Poor	45.2	125,923
Middle	19.7	50,159
Rich	35.1	79,303
Mass media		
No exposure	27.8	72,913
Partial exposure	65.7	165,875
Full exposure	6.5	16,597
Tobacco user		
No	96.7	239,609
Yes	3.3	15,776
Alcohol user		
No	99.5	251,512
Yes	0.5	3873
Region		
North	13.3	47,660
North-East	3.6	36,059
Central	28.0	66,773
East	26.2	50,232
West	12.4	22,328
South	16.5	32,333
Community education		
Low	26.0	68,441
High	74.0	186,944
Ethnic Fractionalization Index		
Totally homogeneous	18.3	63,296
Not homogeneous	81.7	192,089
Religion Fractionalization Index		
Totally homogeneous	65.5	173,928
Not homogeneous	34.5	81,457
Total sample	100	255,385

###  Prevalence of pregnancy loss in India over two time periods

**Figure 1:**
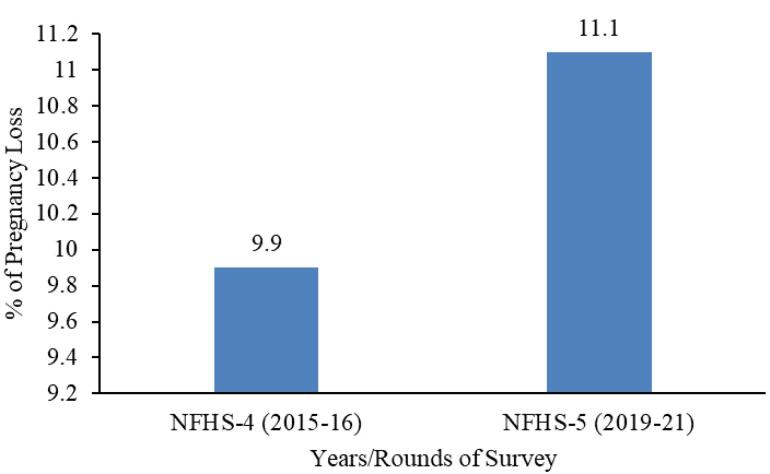
Changes in prevalence of pregnancy losses in India during 2015-2016 to 2019-2021

Figure 1 depicts the prevalence of PL from NFHS-4 to NFHS-5. In the NFHS-5 (2019-2021) survey period, 11.1% of women aged 15-49 at the national level reported experiencing PL, including stillbirth, miscarriage, and abortion. This represents a slight increase compared to the NFHS-4 survey period (2015-2016), indicating a marginal rise in the prevalence of PLs over time.

###  Prevalence of pregnancy loss by district and states

**Figure 2:**
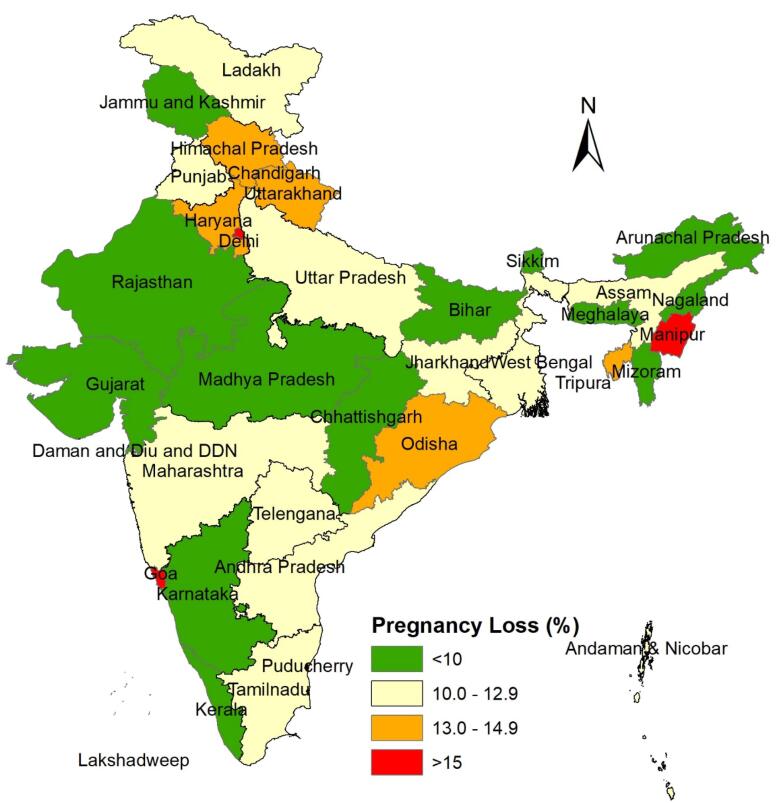
Prevalence of pregnancy losses among women by states in India, 2019-2021

**Figure 3:**
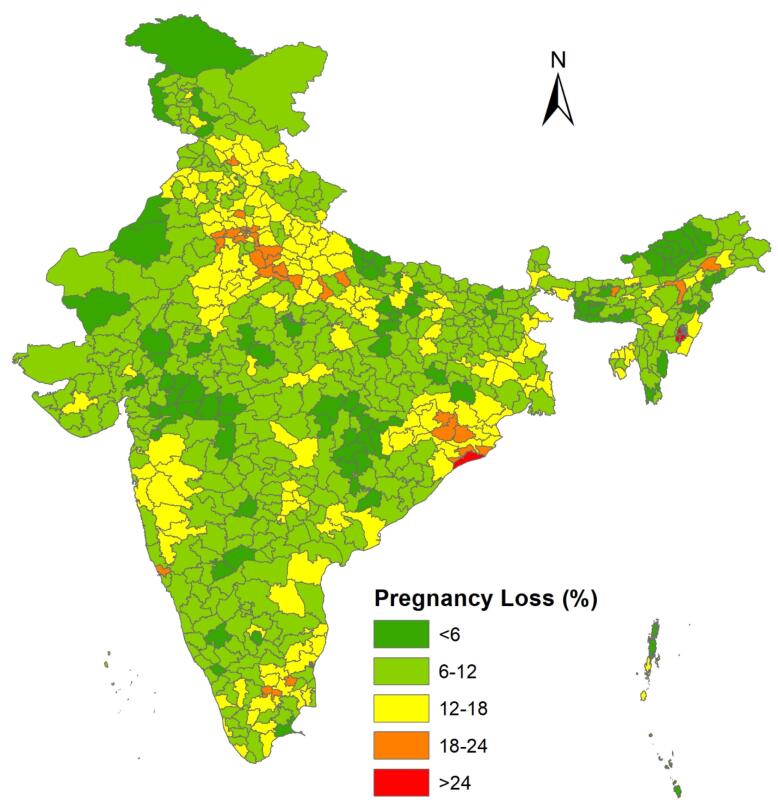
Prevalence of pregnancy losses among women by districts in India, 2019-2021

 The national prevalence of PLs in India is 11.1%, including 7.3% miscarriages, 2.9% abortions, and 0.9% stillbirths. Figure 2 shows the state-wise prevalence of PL in India. In the legend, the green color represents states where the prevalence of PL is less than 10%. Yellow indicates states with a PL prevalence between 10% and 13%, orange shows states with a prevalence between 13% and 15%, and red represents states where the prevalence of PL is greater than 15%.This prevalence varies across the country, ranging from 6.0% in Lakshadweep to 22.9% in Manipur. The highest rates of PLs are found in Manipur, followed by Delhi, Pondicherry, and Goa, while the lowest rates are observed in Lakshadweep, Meghalaya, and Arunachal Pradesh (Figure 2). Similarly, the prevalence of PLs varies by district, ranging from 1.6% in Kra Daadi district of Arunachal Pradesh to 31.4% in the Bishnupur district of Manipur (Figure 3).

###  Prevalence of pregnancy loss by selected exposure variables

Table 2 shows the prevalence of PL by the socio-demographic characteristics of the respondents. The study found a significant positive association between the prevalence of PL and various socio-demographic factors, including women’s age, place of residence, occupation, mass media exposure, tobacco use, region, community education, and the Ethnic Fractionalization Index, with a statistical significance at (χ^2^ *P* value = 0.001).

**Table 2:** Prevalence of pregnancy loss across socio-demographic variables with chi-square analysis, India 2019-2021

**Background**	**Prevalence of PL (%)**	**Number**	***P*** ** value**
Age at the end of pregnancy (y)			0.000
< 20	11.0	29,184	
20-34	10.7	214,354	
35-49	19.2	11,847	
Parity			0.000
One	13.7	67,921	
Two	8.5	95,802	
Three +	6.7	87,078	
Place of residence			0.000
Rural	10.3	201,906	
Urban	13.1	53,479	
Working status			0.162
No	10.6	29,453	
Yes	11.9	9605	
Wealth status			0.000
Poor	9.3	125,923	
Middle	11.9	50,159	
Rich	13.0	79,303	
Mass media			0.000
No exposure	8.7	16,597	
Partial exposure	12.0	165,875	
Full exposure	11.7	72,913	
Tobacco user			0.000
No	11.0	239,609	
Yes	12.9	15,776	
Alcohol user			0.930
No	11.1	251,512	
Yes	12.4	3873	
Region			0.000
North	11.8	47,660	
North-East	11.4	36,059	
Central	11.4	66,773	
East	10.9	50,232	
West	10.7	22,328	
South	10.4	32,333	
Community education			0.000
Low	9.3	68,441	
High	11.7	186,944	
Ethnic Fractionalization Index			0.000
Totally homogeneous	10.1	63,296	
Not homogeneous	11.3	192,089	
Religion Fractionalization Index			0.562
Totally homogeneous	11.1	173,928	
Not homogeneous	11.0	81,457	
Total prevalence for India	11.1	255,385	

*Note.* PL: Pregnancy loss.

 Women aged 35-49 have a higher prevalence of PL (19.2%) compared to younger women. The prevalence of PLs also varies with the number of children women have, with those having only one child experiencing a higher rate of PL (13.7%) than other groups. There is a significant urban-rural difference in PL prevalence, with 13.1% in urban areas and 10.1% in rural areas. Furthermore, working women experience more PLs than non-working women, and wealthier women have a higher prevalence of PL (13%) compared to middle-class (11.9%) and poor women (9.3%).

 Exposure to mass media is also associated with PL. Women with partial (12%) and full (11.7%) mass media exposure are more likely to experience PL than those with no media exposure (8.7%). Additionally, the prevalence of PL is higher among tobacco users (12.9%) and alcohol users (12.4%). The prevalence of PL varies by region, with higher rates observed in the North, North-East, and Central parts of India compared to other regions. Community education is significantly associated with PL prevalence, with higher levels of maternal education correlating with higher rates of PL. The Ethnic Fractionalization Index indicates that higher levels of ethnic and religious diversity may slightly increase the prevalence of PL.

###  Spatial pattern of pregnancy loss

**Figure 4:**
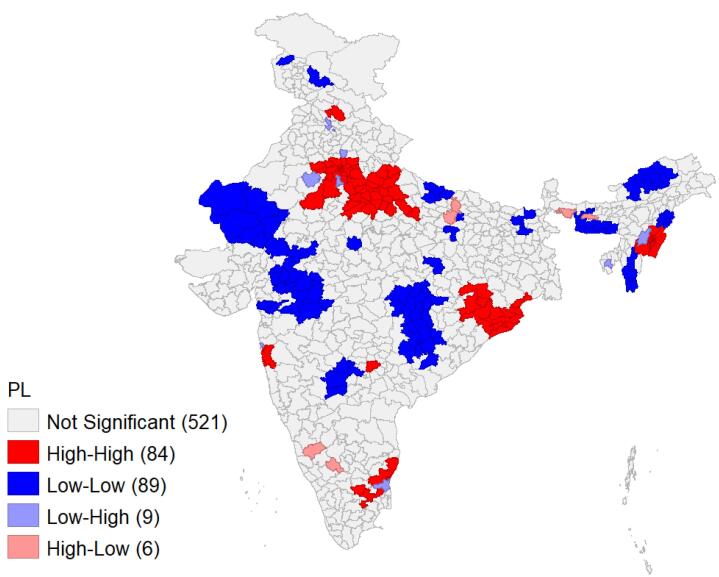
Univariate LISA Cluster Showing Spatial Clustering and Outliers of PL (Moran’s I = 0.559) *Note.* LISA: Local indicator of spatial association; PL: Pregnancy loss

**Figure 5:**
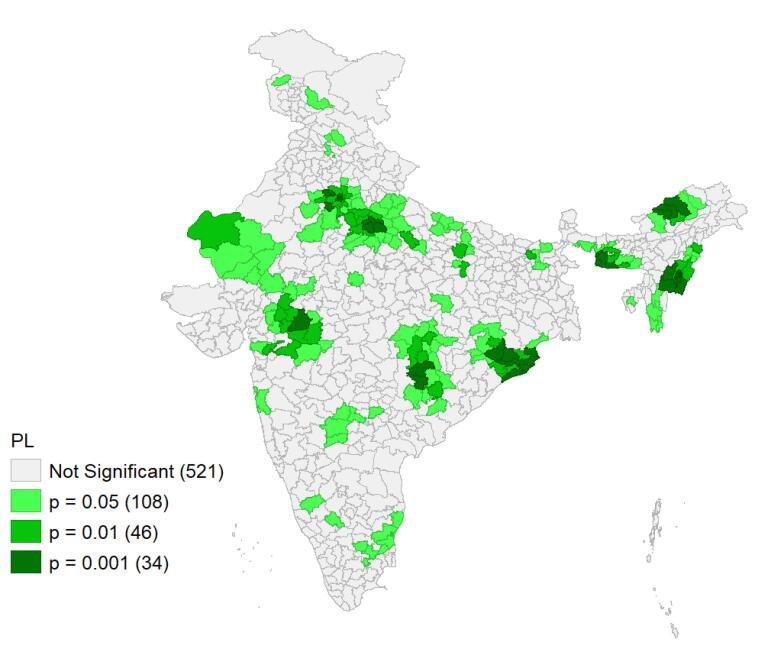
Significance map for pregnancy loss showing significant locations among indian districts

 The univariate Moran’s I index value for PL, which was 0.56, indicates a substantial degree of spatial autocorrelation across the districts in India. The univariate LISA cluster map for PLs in Figure 4 illustrates the spatial clustering of PL by district in India, while Figure 5 presents the significance map, highlighting areas significant at the 5% level. In the map legend, five colors are used: the white color indicates non-significant districts, the red color represents districts with high-high values (hot spots)- districts with above-average prevalence of PLs- which share boundaries with neighboring districts also having above-average values, and the blue color indicates districts with low-low values (cold spots). In Figure 4, 84 districts with significant hot spots were identified, indicating a high prevalence of PLs, primarily located in Delhi, Uttar Pradesh, Manipur, Odisha, Haryana, Rajasthan, and some districts of Tamil Nadu, Pondicherry, Goa, and Maharashtra. Conversely, 89 districts were identified as cold spots, showing lower prevalence of PL, in regions such as Madhya Pradesh, Rajasthan, Chhattisgarh, Maharashtra, Arunachal Pradesh, Jammu and Kashmir, and Mizoram. Additionally, six districts exhibited a high prevalence of PL despite being surrounded by districts with low prevalence. Nine districts with a low-high pattern were identified, characterized by a low prevalence of PL surrounded by districts with high prevalence. These can be considered positive deviant districts.

###  Risk factors of pregnancy loss

####  Fixed effect model

Table 3 presents the results from multilevel models examining the association between various exposure variables and PL. Significant positive associations were found for women’s age, parity, working status, place of residence, mass media exposure, wealth status, tobacco use, region, and Ethnic Fractionalization Index with PL.

**Table 3:** Results for the individual and community-level factors linked to PL in India, 2019-2021

** Background variables**	**Model 2**
	**Adjusted OR (95% CI)**
Fixed effect part	
Age at the end of pregnancy	
< 20	1.00
20-34	1.43 (1.26, 1.63)
35-49	3.80 (3.15, 4.58)
Parity	
1	1.00
2	0.60 (0.55, 0.65)
≥ 3	0.39 (0.35, 0.43)
Place of residence	
Rural	1.00
Urban	1.15 (1.04, 1.26)
Working status	
No	1.00
Yes	1.17 (1.07, 1.27)
Wealth status	
Poor	1.00
Middle	1.20 (1.08, 1.33)
Rich	1.20 (1.08, 1.34)
Mass media	
No exposure	1.00
Partial exposure	1.19 (1.02, 1.38)
Full exposure	0.98 (0.83, 1.17)
Tobacco user	
No	1.00
Yes	1.28 (1.09, 1.49)
Alcohol user	
No	1.00
Yes	1.23 (0.93, 1.63)
Region	
South	1.00
North	1.20 (1.03, 1.40)
North, East	1.11 (0.93, 1.33)
Central	1.26 (1.08, 1.47)
East	1.56 (1.32, 1.83)
West	0.97 (0.80, 1.18)
Community education	
Low	1.00
High	1.03 (0.93, 1.13)
Ethnic Fractionalization Index	
Totally homogeneous	1.00
Not homogeneous	1.19 (1.08, 1.32)
Religion Fractionalization Index	
Totally homogeneous	1.00
Not homogeneous	1.02 (0.94, 1.11)
Random effect part	
District	0.095 (0.06, 0.13)

*Note.* PL: Pregnancy loss; OR: Odds ratio; CI: Confidence interval. Model 1 (empty model, not shown in this table) contains no exposure variables, decomposing total variance at the district level. Model 2 contains exposure variables at both the individual and contextual levels.

 Women aged 35-49 years were 3.8 times more likely to experience PLs compared to younger women under 20 years old (aOR = 3.8, 95% CI: 1.26-1.63). Parity was negatively associated with PLs, indicating that women with more than one child had a lower risk of experiencing these outcomes. Urban women had a 1.2 times higher risk of PLs (aOR: 1.2, 95% CI: 1.04-1.26) compared to those in rural areas.

 Working women were 1.2 times more likely to have PLs (aOR: 1.17, 95% CI: 1.07-1.27). Women from the rich (aOR: 1.20, 95% CI: 1.08-1.33) and middle-class (aOR: 1.20, 95% CI: 1.08-1.34) categories had a significantly higher risk compared to poor women. Partial media exposure was associated with a higher risk of PL (aOR: 1.19, 95% CI: 1.02-1.38). Additionally, women who used tobacco exhibited a higher risk of PL (aOR: 1.28, 95% CI: 1.09-1.49). Women aged 35-49 years were 3.8 times more likely to experience PLs compared to younger women under 20 years of age (aOR = 3.8, 95% CI: 1.26-1.63)

 Regionally, women from the North (aOR: 1.20, 95% CI: 1.03-1.40), East (aOR: 1.56, 95% CI: 1.32-1.83), and Central (aOR: 1.26, 95% CI: 1.08-1.47) regions experienced a higher risk of PL compared to their counterparts. Although community education was not significantly associated with PL, higher odds were observed for more educated women. The Ethnic Fractionalization Index was positively associated with PL, with lower community-level ethnic and religious concentration significantly increasing the likelihood of these losses among women.

####  Random effect model

 The null model, or empty model, does not include any exposure variables and is used to decompose the total variance at the district level (not shown in the able). This model helps determine the extent to which individual-level variation is attributed to the district level. The VPC of the null model was 5.1%, indicating that approximately 5.1% of the variation in PL is due to the clustering effect at the district level. After adjusting for individual and contextual factor, the VPC value decreased to 2.8%, suggesting that the remaining variation in PL is attributed to differences among respondents at the individual, household, and other unknown factors (not shown in the table). A high VPC value signifies significant clustering of PL across districts, as well as a strong neighborhood effect on individual risk.

 Additionally, the proportion change in variance value in the full model was 0.464, indicating that 46.4% of the variation in PLs can be explained by individual and contextual factors.

## Discussion

 The study presented the prevalence of PL, its spatial pattern, and clustering across different districts and identified risk factors for PL in India using multilevel analysis. The overall prevalence of PL in India was 11%, which is higher than in many less developed countries such as the Democratic Republic of the Congo (2.9%), Ghana (4.9 %), and Zambia (0.8%).^21,26^ An Ethiopian study found higher PL rate of 13.9%.^1^ In India, the highest rates of PL were found in Manipur, followed by Delhi, Pondicherry, and Goa, while the lowest rates were observed in Lakshadweep, Meghalaya, and Arunachal Pradesh. These results align with a recent study by Swain et al using the data from NFHS-5 to analyze the pattern and trend of PL during 1992-2021.^7^

 The study also highlighted the spatial pattern of PL by districts, identifying 84 districts as hot spots with a high prevalence of PL. These major hot spots were found mainly in Northern and Eastern Indian states such as Manipur, Delhi, Uttar Pradesh, Haryana, Rajasthan, Odisha, and some districts in Tamil Nadu, Pondicherry and Maharashtra. Cold spots were observed in districts from the states of Madhya Pradesh, Rajasthan, Chhattisgarh, Maharashtra, Andhra Pradesh, Jammu and Kashmir, and Mizoram. These hot spots reflect socio-cultural differences, economic inequality, health system barriers, and variations in women’s health conditions.

 Socio-cultural barriers such as teenage and delayed pregnancies, maternal age, mode of conception, and psychological well-being of women can affect pregnancy outcomes.^27,28^ Moreover, high PL rates are influenced by medical factors, including anemia, infections, hypertension, hyperglycemia, spousal violence, and environmental pollution.^17,29^ Furthermore, economic inequality across states and rural-urban areas, varied levels health funding, and accessibility to healthcare services lead to the variation in PL prevalence.^30^

 The study also assessed the risk factors for PL in India. Significant positive associations were found between PL and factors such as women’s age, parity, working status, place of residence, mass media exposure, wealth status, tobacco use, region, and Ethnic Fractionalization Index. Regarding maternal age, our study found that women aged 35-49 years were more likely to experience PL compared to younger women under 20, similar to previous studies in the literature.^8,10,13^ Parity was negatively associated with PL, indicating that women with more than one child had a lower risk of experiencing PL.

 Urban residents and women from higher wealth indices were at a greater risk of experiencing PL compared to their rural counterparts and women from lower wealth indices, consistent with the results of the studies that assessed PL risk factors using NFHS-4 data.^7,8^ This can be due to a more sedentary lifestyle observed most commonly among urban and wealthier populations. Despite greater access to health services, this lifestyle can induce various health problems such as diabetes, and hypertension, which increase the likelihood of experiencing PL.

 In our study, exposure to mass media was also associated with PL. Women with ‘partial’ and ‘full’ mass media exposure were more likely to experience PL compared to women with ‘no’ media exposure. Although existing literature shows that increased mass media consumption by mothers can increase health service utilization and improve health outcomes,^31,32,33^ the findings of our study vary from the existing literature in this regard. Some studies suggest that women with media exposure may experience greater self-efficacy in making informed decisions about abortion.^28^ Some literature highlights the complex relationship between media exposure and public health outcomes. While media can raise awareness about health issues, it does not always lead to positive outcomes.^34^ Furthermore, other studies have highlighted that excessive use of social media is associated with reduced physical activity and poor sleep patterns, which may contribute to APOs.^35^ Women who used tobacco were at a higher risk of PL, a finding consistent with existing literature.^8,11,36,37^

 The spatial distribution of PL shows that women from the North, East, and Central regions of India had a higher risk of PL compared to women from other regions. However, the literature presents mixed results for this variable, based on the same dataset. A study by Swain et al showed that PL risk was higher in Eastern India compared to other regions.^10^ Conversely, another study assessing the pattern and trends from 1990 to 2021 reported an increased risk of PL in South India as well.^7^

 While no statistically significant correlation was found between community education and PL, women with higher levels of education exhibited a higher likelihood of experiencing PL. Additionally, unfavorable pregnancy outcomes were strongly associated with the Ethnic Fractionalization Index, with a significantly increased likelihood of these outcomes in communities with lower ethnic and religious concentration.^38^ This finding is consistent with existing literature, emphasizing that ethnic diversity within a community may lead to unequal access to healthcare, which may result in poor health outcomes.^39^ Furthermore, research indicates that ethnic and racial disparities continue to worsen or persist in fetal, neonatal, and maternal health outcomes.

**Highlights**

- Pregnancy loss (PL) prevalence was 11.1 % in 2019-2021, which rose from 9.9% in 2015-2016
- Higher PL rates correlate with older maternal age, urban residence, higher wealth index, and tobacco use.
- Hot spots for PL were primarily located in the Northern and Eastern states of India.

## Conclusion

 This study highlights the high prevalence of PL in India while identifying key risk factors using nationally represented data. PL serves as an excellent tool for assessing the effectiveness of MCH programs and acts as an indicator of MCH services within the country. Despite India’s ongoing efforts to improve MCH, the prevalence of PL has increased from 2015-2016 to 2019-2021. This upward trend of PL calls for equitable and targeted interventions to improve the quality and uptake of MCH services across India. By identifying regional variations in PL prevalence and understanding the contributing risk factors, this study can provide insights for developing targeted interventions and deliver ‘what is needed’ to ‘where it is needed’. Studies like these will help channel national resources aimed at strengthening MCH and yielding better outcomes in the long run. A region-wise approach to improving health indicators is recommended to achieve favorable health outcomes.

## Acknowledgments

 The authors would like to express their sincere gratitude to the Director of ICMR-National Institute for Research in Reproductive and Child Health, Mumbai, and the Additional Director General for their comments and support during this study.

## Competing Interests

 The authors declare no conflict of interests.

## Ethical Approval

 This study received ethical approval from ICMR-NIRRCH (IEC No: D/ICEC/Sci-35/39/2022, dated 27/04/2022). The study utilized publicly available secondary data published by the National Family Health Survey, the Government of India, and the Demographic and Health Surveys Program. Consent was obtained from the study participants by the respective organizations. Therefore, obtaining consent for participation in our research was not applicable.

## Funding

 No funding was received for this research.
